# Diabetes medication use in the last year of life in people living with dementia in the United Kingdom and Australia

**DOI:** 10.1007/s41999-026-01608-6

**Published:** 2026-09-27

**Authors:** Darshna Goordeen, Olivia K. Hopkinson, Emily Reeve, Joshua D. Niznik, Wallis C. Y. Lau, Li Wei, Jenni Ilomaki, J. Simon Bell

**Affiliations:** 1 Centre for Medicine Use and Safety, Faculty of Pharmacy and Pharmaceutical Sciences, Monash University, 381 Royal Parade, Parkville, VIC 3052 Australia; 2 Research Department of Practice and Policy, School of Pharmacy, University College London, London, UK; 3 School of Pharmacy and Biomedical Science, Adelaide University, Adelaide, South Australia Australia; 4 Eshelman School of Pharmacy, University of North Carolina, Chapel Hill, North Carolina USA; 5 Centre for Medicines Optimisation Research and Education, University College London Hospitals NHS Foundation Trust, London, UK; 6 Centre for Safe Medication Practice and Research, Department of Pharmacology and Pharmacy, Li Ka Shing Faculty of Medicine, University of Hong Kong, Hong Kong, China; 7 Laboratory of Data Discovery for Health (D24H), Hong Kong Science Park, Pak Shek Kok, Hong Kong, China

**Keywords:** Type 2 diabetes, Dementia, Antihyperglycaemics, End-of-life care

## Abstract

**Purpose:**

To investigate the prevalence of type 2 diabetes (T2D) medication use in the final 3,6, 9, and 12 months of life and explore patient factors associated with discontinuation in the 6 months prior to death in people living with dementia in the UK and Australia.

**Methods:**

Linked hospital and administrative data were used from the UK and Australia. We included individuals who died between July 2013 and June 2018. Logistic regressions estimated the association between age, sex, cardiovascular disease, chronic kidney disease (CKD), and cancer with discontinuation of T2D medications.

**Results:**

10,088 UK and 6,436 Australian individuals were identified. From those supplied insulin in the last 12 months of life, there was a 25.6% reduction in use in the UK and 43.5% in Australia in the last 3 months of life, but minimal reduction in other medication classes. CKD was associated with metformin discontinuation in the last 6 months of life in the UK (OR 1.05, 95% CI 1.03–1.07) and Australia (OR 1.60, 95% CI 1.15–2.12).

**Conclusion:**

Guideline-concordant discontinuation of metformin in the last 6 months of life was observed in those with CKD. Decreased insulin supply in those living with dementia was observed. There were lower rates of discontinuation of other glucose-lowering medications in the UK and Australia.

**Supplementary Information:**

The online version contains supplementary material available at https://doi.org/10.1007/s41999-026-01608-6.

## Key summary points

**Aim:**

To investigate the use of glucose-lowering medication for Type 2 Diabetes (T2D) in the final 12 months-of-life in people living with dementia in the UK and Australia.

**Findings:**

Glucose-lowering medications were more likely to be discontinued in the last year of life in people living with dementia in Australia compared to the UK.

Insulin was the medication class most likely to be discontinued in the UK and Australia.

**Message:**

For people living with dementia and T2D in the UK and Australia, use of glucose lowering medications often persisted until death, highlighting the continued need for promotion of patient-centred deprescribing pathways to improve comfort and reduce potential harm.

**Supplementary Information:**

The online version contains supplementary material available at https://doi.org/10.1007/s41999-026-01608-6.

## Background

The United Kingdom (UK) and Australia have recognised the need for improving end-of-life care for people living with dementia through national dementia strategies and action plans [1, 2]. Dementia clinical practice guidelines highlight the need for individualising treatment according to changing treatment goals when life expectancy is limited [3–5]. Dementia and type 2 diabetes (T2D) often co-exist in older adults, predisposing them to a decline in health status, frailty and vulnerability to adverse drug events (ADEs) (e.g. hypoglycaemia).

Whilst tight glycaemic control is recommended for adults, guidelines in the UK and Australia recommend less strict control (e.g. higher glycated haemoglobin [HbA1c] targets) in those living with dementia and limited life expectancy [4, 6]. Poor nutrition and inability to manage complex treatment regimens are common with advanced dementia. This can increase the risk of medication-related harm due to errors in medication administration, irregular eating habits and reduced ability to recognise, report or treat hypoglycaemia [7, 8]. Deprescribing of glucose-lowering medication may be warranted for individuals with dementia and T2D [9]. Deprescribing is defined as the planned and supervised process of dose reduction or stopping of medication that might be causing harm, or no longer be of benefit [10]. Deprescribing and medication regimen simplification may include medication discontinuation, switching to a safer alternative, dose reduction or consolidation of administration times.

Despite general acceptance that deprescribing can be safe and feasible [11], people living with dementia are often overtreated with glucose-lowering medication. A retrospective study investigating glucose-lowering medication in those with dementia found that amongst 161 patients, 54% of the cohort were potentially overtreated (HbA1c under 7% for those with mild functional cognitive impairment, or multiple co-morbidities and 7.6% for significant functional or cognitive impairment) [12]. Rates of potential overtreatment with glucose-lowering medications in aged care homes range from 5–86% [13]. These studies highlight the value of investigating medication supply in the last year of life when potential harms may outweigh potential benefit.

The objective of this study was to investigate the prevalence of glucose-lowering medication use in the final 3, 6, 9 and 12 months of life in people living with dementia and to explore patient factors associated with discontinuation in the 6 months prior to death in the UK and Australia.

## Methods

This multi-national study was a retrospective observational analysis of linked hospital and administrative data to evaluate diabetes medication use in the last year of life in people living with dementia in UK and Australia. This study was approved by the Australian Institute of Health and Welfare Ethics Committee (approved amendment to EO2018/4/468), Monash University Human Research Ethics Committee (Project ID 14339), and the use of UK data was approved via Clinical Practice Research Datalink (CPRD)’s Research Data Governance Process (ref: 23_002757).

### Data sources

The CPRD is a UK-based service sponsored by the Medicines and Healthcare Products Regulatory Agency and the National Institute for Health and Care Research, as part of the Department of Health and Social Care. CPRD GOLD and Aurum are primary care databases comprising anonymised primary care data from general practices across the UK to provide a longitudinal, representative UK population health dataset. Data on patient demographics (age and sex) and prescriptions were used from CPRD [14, 15]. Linked data from the Office for National Statistics were used to provide information on patient death status and linkage with Hospital Episodes Statistics identified dementia, cardiovascular disease (CVD), cancer, and chronic kidney disease (CKD) diagnoses made in secondary care.

In Australia, we analysed linked administrative data from the Victorian Admitted Episodes Dataset (VAED), Pharmaceutical Benefits Scheme (PBS), and National Death Index (NDI). The VAED was used for demographics and diagnoses. PBS data included medication name and strength, dispensed quantity and date of supply for medications subsidised by the Australian Government. This dataset was used to determine supply of glucose-lowering and dementia medications. The NDI was used to ascertain each person’s date of death. We analysed data from 1 July 2012 to 30 June 2018 to correspond with the availability of under patient co-patient PBS dispensing records.

### Study design and study sample

**Fig. 1:**
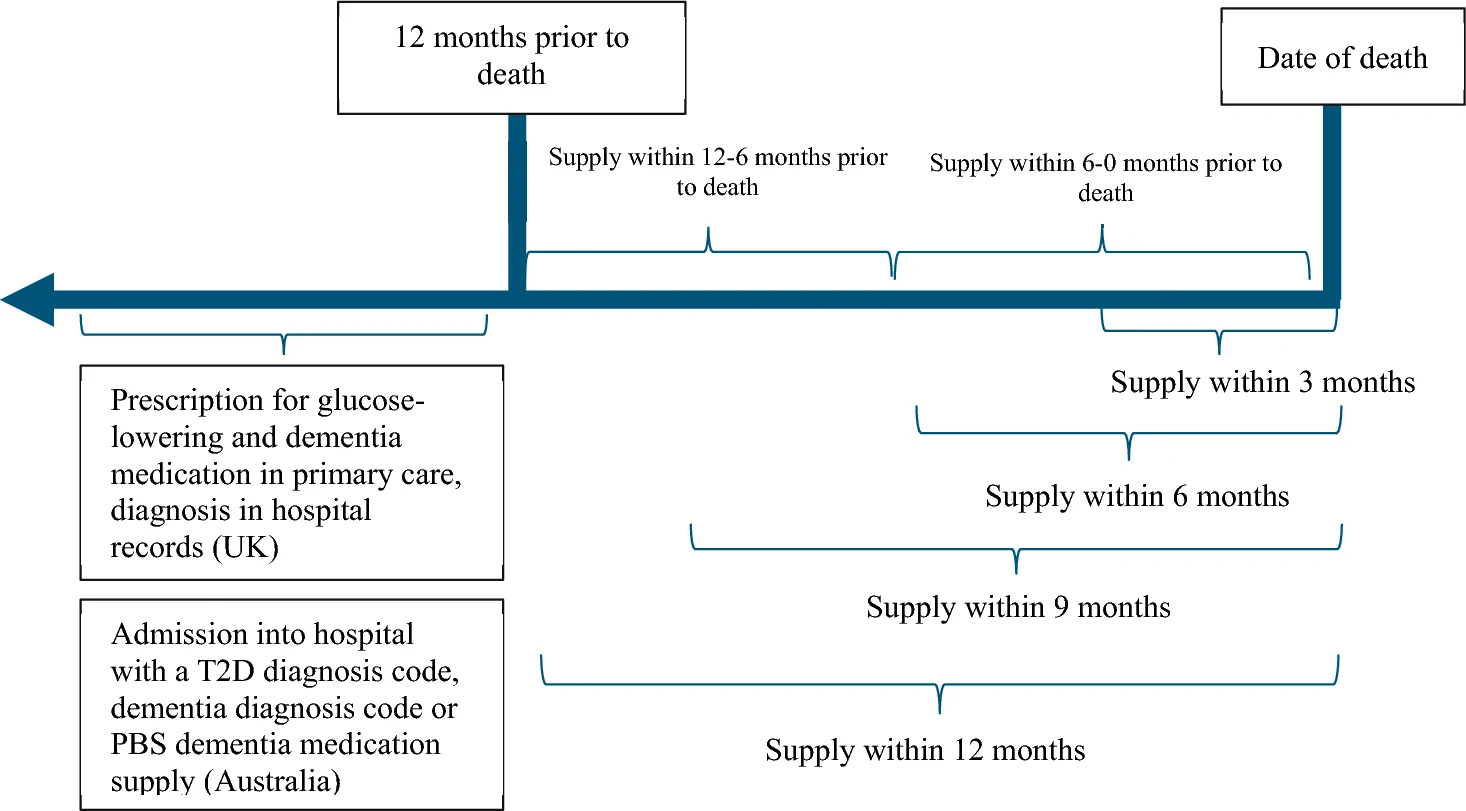
Study timeline

The study population included adults with a recorded death between 1 July 2013 and 30 June 2018 and diagnosed with both T2D and dementia at least 12 months prior to death in the UK or Victoria, Australia. Glucose-lowering medications included insulin, metformin, sulfonylureas and other glucose-lowering medications (dipeptidyl peptidase−4 (DPP-4i), sodium-glucose co-transporter 2 inhibitors (SGLT-2i), glucagon-like peptide−1 receptor agonist (GLP-1ra), thiazolidinediones (TZDs)). The index date was defined as the date of death and the assessment period was defined as 12 months prior to death. In the UK, the earliest date of prescription of glucose-lowering medication (categorised using British National Formulary) or diagnosis in hospital records/hospital episode statistic linkage was used to identify individuals with T2D. Similarly, people with dementia were identified at the earliest date of prescription of dementia-related medication or a diagnosis of dementia being recorded in hospital records via ICD-10 codes. In Australia, people with T2D were identified through the first entry with ICD-10 codes for T2D in the VAED recorded least 12 months prior to death. Similarly, dementia was identified through ICD-10 codes in VAED at least 12 months prior to death or a PBS claim for dementia medication at least 12 months prior to death (Fig. 1). In the UK, we reported the age at the death and baseline co-morbidities were captured at any time prior to the assessment period. In Australia, sex and age group were captured at the first hospital admission when a T2D diagnosis code was recorded during the study period at least 12 months prior to death. Co-morbidities were captured at any hospital admission until the assessment period and classified according to ICD-10 diagnosis codes and medications by the Anatomical Therapeutic Chemical (ATC) codes (Supplementary Information Table 1a, b, c). Discontinuation was defined as no prescription in the UK or dispensing in Australia of each medication class during the final 9, 6, or 3 months of life for the prevalence analyses, and during the final 6 months of life for the regression analyses.

**Table 1:** Cohort characteristics

	Australia		United Kingdom	
Variable	Frequency (*N*=6436)	Percent (%)	Frequency (*N*=10,088)	Percent (%)
Sex				
Male	3153	49	4148	41.1
Female	3283	51	5940	58.9
Age*				
≤ 74	1304	20.3	711	7.0
75–84	2709	42	3747	37.1
85+	2423	37.7	5630	55.8
Comorbidities				
Cardiovascular disease	5339	83	9200	91.2
Chronic kidney disease	4181	64.5	3529	35.0
Cancer	1842	28.6	2091	20.7

^*^Age at first hospital admission with type 2 diabetes for Australia; age at 1 year prior to death for the UK

### Statistical analysis

Summary statistics were used to describe age, sex and co-morbidities and prevalence of glucose-lowering medications at cumulative quarterly intervals (Fig. 1). These were presented as frequencies and percentages for categorical variables. Prevalence was calculated based on having medication supplied within each time period (i.e. the last 3, 6, 9 and 12 months of life). We also compared the prevalence across time periods.

Separate regression models were fitted to investigate the likelihood discontinuation by medication class in the last 6 months before death amongst those supplied glucose-lowering medication at least once in 6 to 12 months prior to death (i.e. amongst current users who had not discontinued) (Fig. 1). Covariates included age group, sex, and selected comorbidities at baseline (cardiovascular disease, chronic kidney disease and cancer). Logistic regression models were applied, with results presented as odds ratios (ORs) and 95% confidence intervals (CIs) to determine the likelihood of discontinuation of glucose-lowering medication in the final 6 months of life. Model fit and assumptions were evaluated to ensure robustness.

Analyses were conducted using the statistical software package SAS version 9.4 (SAS Institute Inc., Cary, NC, United States), R Statistical Software (version 4.2.2) and Microsoft Excel.

This work was supported by the PharmAlliance Research Cluster for Doctoral Training scholarship between Monash University, University College London and University of North Carolina, Chapel Hill. They played no role in the design, execution, analysis and interpretation of data, or writing of the study.

## Results

### Study characteristics

**Fig. 2:**
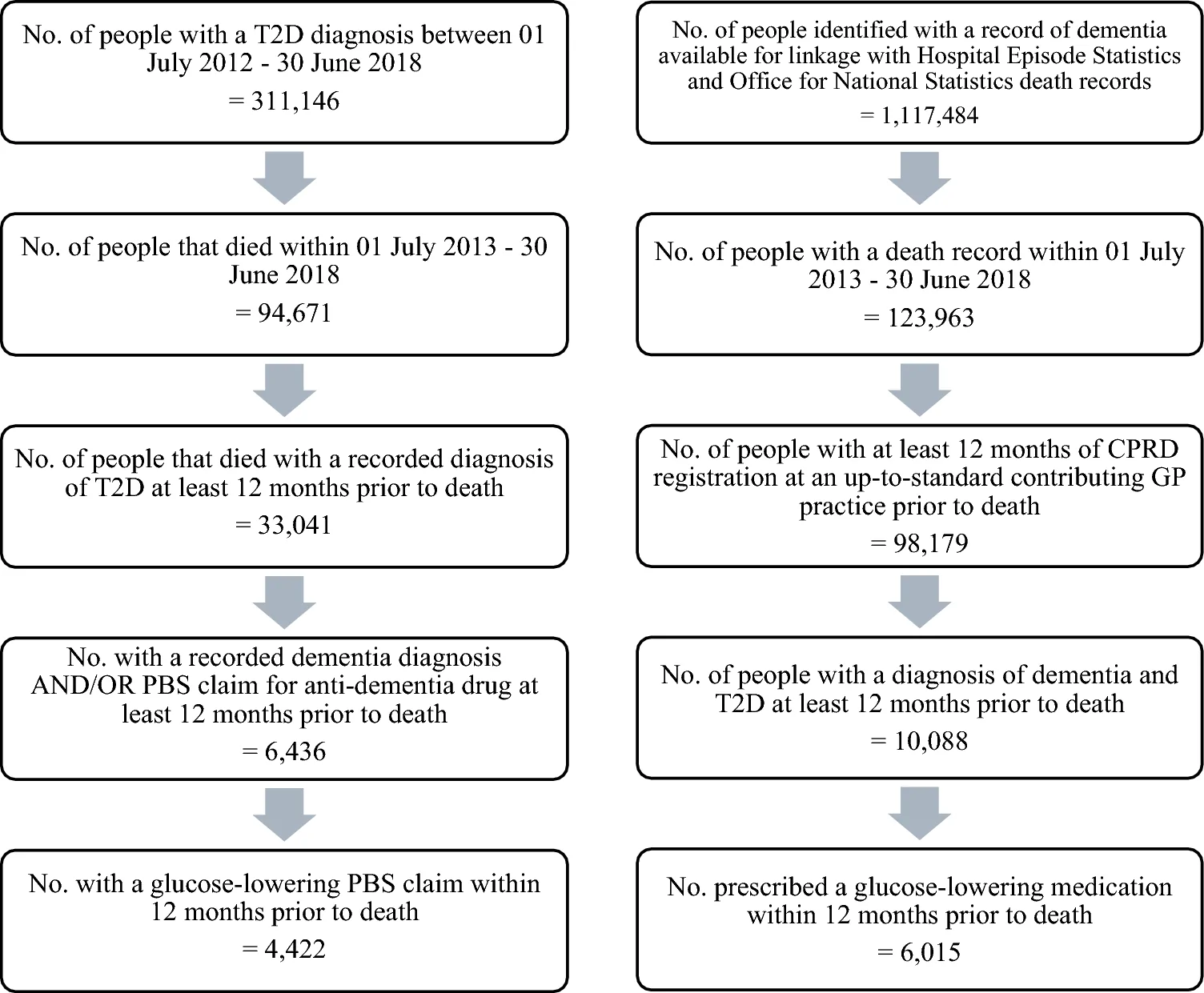
Selection of cohort

There were 10,088 UK and 6,436 Australian individuals with T2D and dementia who died at least 12 months after a recorded diagnosis (Fig. 2). Both comprised over 50% females (58.9% UK, 51% Australia). Overall, 55.8% and 37.7% of individuals were aged 85 years or older in the UK and Australia, respectively. There was a higher documented prevalence of CVD in the UK than in Australia (91.2% versus 83%) but a lower documented prevalence of CKD (35% versus 64.5%) (Table 1).

### Prevalence of use in the last year of life

During the last 12 months of life, metformin was the most commonly prescribed or dispensed glucose-lowering medication class in both countries. In the UK and Australia, use was 36% and 41% for metformin, 19.5% and 33.8% for insulin, 21.6% and 27.9% for sulfonylureas and 11.2% and 15.1% for other glucose-lowering agents (DPP-4is, SGLT-2is, GLP-1ras, and TZDs) (Supplementary Information Table 2).

**Table 2:** Likelihood of glucose-lowering medication supply in the last 6 months of life

	Metformin				Insulin				Sulfonylureas				Other glucose-lowering medications			
	Australia		UK		Australia		UK		Australia		UK		Australia		UK	
Variable	OR	95% CI	OR	95% CI	OR	95% CI	OR	95% CI	OR	95% CI	OR	95% CI	OR	95% CI	OR	95% CI
Age ≤74 vs 85+	0.89	0.63–1.27	1.02	0.98–1.05	0.77	0.57- 1.02	0.98	0.94–1.03	1.67	1.16–2.40	1.08	1.02–1.14	0.82	0.27–2.51	0.94	0.89–0.99
Age 75–84 vs 85+	1.07	0.80–1.43	0.997	0.98–1.02	0.89	0.69–1.16	0.99	0.97–1.03	1.17	0.87–1.58	1.02	0.99–1.05	1.91	0.65–5.62	0.98	0.95–1.01
Male vs female	0.73	0.56–0.93	0.98	0.97–1.00	1.17	0.93–1.45	0.99	0.96–1.02	0.76	0.59–1.00	0.996	0.97–1.03	1.60	0.75–3.41	0.96	0.93–0.99
CVD vs no CVD	1.10	0.78–1.57	1.02	0.98 − 1.05	0.60	0.43–0.84	0.95	0.90–1.01	1.07	0.74–1.56	1.01	0.96–1.06	1.10	0.38–3.20	1.00	0.93–1.07
CKD vs no CKD	1.60	1.22–2.12	1.05	1.03–1.07	0.85	0.65–1.12	0.10	0.97–1.05	1.07	0.80–1.45	1.01	0.98–1.04	1.46	0.62–3.45	0.97	0.93–1.01
Cancer vs no cancer	1.06	0.81–1.40	0.994	0.97–1.02	0.89	0.69–1.14	1.01	0.97–1.05	1.10	0.83–1.46	0.99	0.95–1.02	0.78	0.36–1.70	0.97	0.93–1.01

*OR* odds ratio, *CI* confidence interval, *CVD* cardiovascular disease, *CKD* chronic kidney disease

**Fig. 3:**
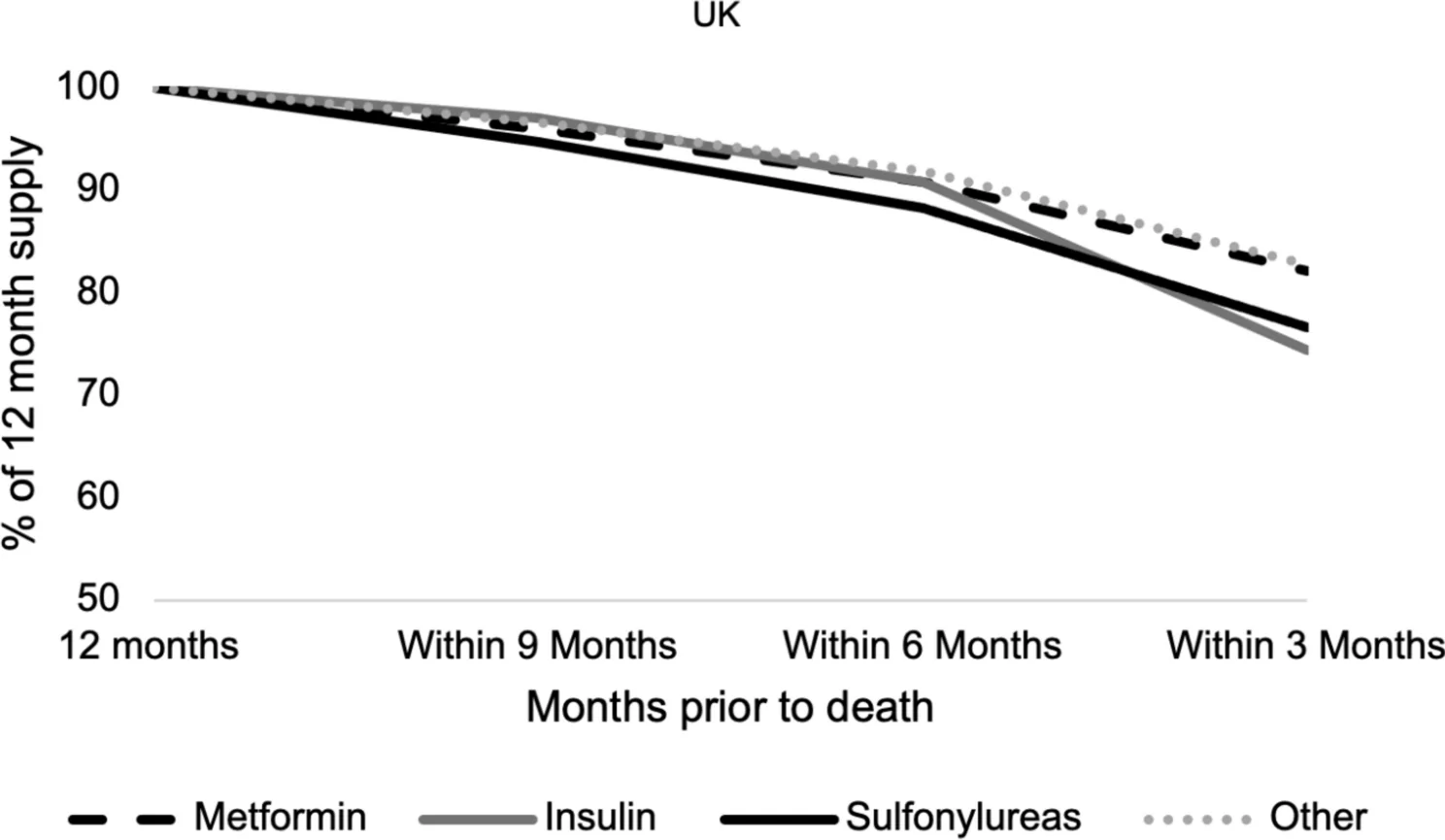
Prevalence of glucose-lowering medication supply in the last 12 months of life in UK. Caption: Percent of 12-month prescriptions within 12, 9, 6 and 3 months prior to death in the UK shows minimal decline with insulin and sulfonylureas falling the most. At 3 months prior to death, metformin= 82.1%, insulin = 74.4%, sulfonylureas= 76.6% and other= 82.8% of 12-month prescriptions. Y-axis 50–100%

**Fig. 4:**
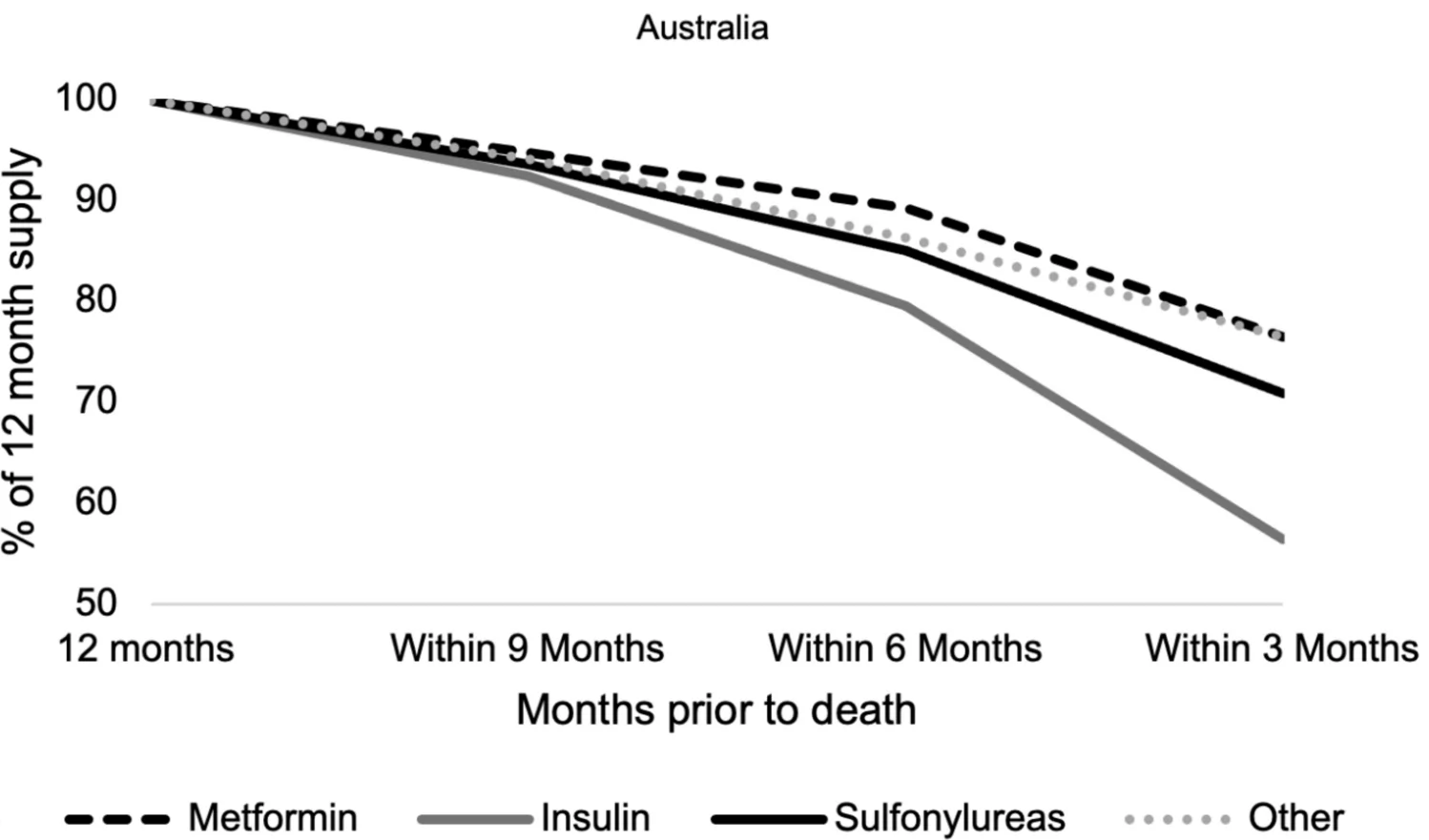
Prevalence of glucose-lowering medication supply in the last 12 months of life in Australia, Caption: Percent of 12-month supply within 12, 9, 6 and 3 months prior to death in Australia shows a slight decline across glucose-lowering medication classes, with a marked fall for insulin. At 3 months prior to death, metformin= 76.5%, insulin= 56.5%, sulfonylureas= 70.9%, other= 76.5% of 12 month. Y-axis 50–100%

In both the UK and Australia, prevalence of medication supply generally declined as the time of death approached (Fig. 3, 4). In the UK, of the individuals prescribed insulin in the 12 months prior to death, 97.1% received a prescription within 9 months, 90.8% in 6 months and 74.4% in their final 3 months. For sulfonylureas, 94.7% of those with prescriptions within 12 months had prescriptions within the last 9 months, 88.3% in 6 months and 76.6% in the last 3 months. In Australia, insulin supply showed a more pronounced difference with 92.5% being supplied within 9 months, 79.6% within 6 months and only 56.5% within the final 3 months. Supply of other glucose-lowering medications (DPP-4is, SGLT-2is, GLP-1ras, TZDs) was observed as 94.1%, 86.3% and 76.5% of 12-month users receiving supply within 9, 6 and 3 months, respectively (Supplementary Information Table 2).

### Discontinuation of diabetes medication in the last 6 months of life

Among those supplied with a glucose-lowering medication within 6 to 12 months prior to death, a 9.4–26.9% absolute reduction in use was observed in the last 6 months of life (Supplementary Information Table 3). In the UK, there was a lower proportion of patients supplied sulfonylureas in the last 6 months, with 12.3% not receiving supply within the last 6 months of life. In Australia, 26.9% of individuals were not supplied insulin in the last 6 months of life.

### Metformin

Chronic kidney disease (CKD) in the UK was associated with slightly higher odds of discontinuation of metformin (OR 1.05, 95% CI 1.03–1.07) in the last 6 months of life. In Australia, CKD was also associated with higher odds of discontinuation (OR 1.60, 95% CI 1.15–2.12) and male sex was associated with lower odds of discontinuation (OR 0.73, 95% CI 0.57–0.94) (Table 2).

### Insulin

No covariates were associated with discontinuation of insulin in the UK. In Australia, CVD was associated with reduced odds of discontinuation of insulin (OR 0.6, 95% CI 0.43–0.84) (Table 2).

### Sulfonylureas

Being aged **≤**74 years in the UK and Australia was associated with higher odds of discontinuation compared to those aged 85 years and older (OR 1.08, 95% CI 1.02–1.14) (OR 1.67, 95% CI 1.16–2.34). Other factors, such as sex, CVD, CKD and cancer, did not show associations (Table 2).

### Other glucose-lowering medications

Females in the UK had slightly higher odds for discontinuation of other glucose-lowering medications in the last 6 months of life (OR 0.96, 95% CI 0.93–0.99) compared to males as well as those aged **≤**74 years compared to those aged 85 years and older (OR 0.94, 95% CI 0.89–0.99). No variables showed association with discontinuation in the Australian cohort (Table 2).

## Discussion

This was the first study to examine glucose-lowering medication use in the last year of life of people living with dementia in the UK and Australia. Our study showed a 9.4–26.9% reduction in medication use in the last 6 compared to last 12 months of life with the magnitude of discontinuation varying between medication classes and countries. These findings suggest inadequate discontinuation of glucose-lowering medications in the last year of life. However, CKD was associated with discontinuation of metformin in the last 6 months of life. This suggests discontinuation reflects consideration of specific patient co-morbidities.

Insulin use declined in the last 12 months of life in both the UK and Australia. This was in contrast to Danish and US studies that have reported stable or increased insulin use during the final year of life amongst older adults and those classified as frail [16, 17]. However, these studies did not report data specific to people living with dementia. Our observed insulin discontinuation likely reflects that current practice encompasses clinical considerations required when managing T2D in people living with dementia. These include the associated risks of insulin use in this population such as difficulty with administration, diminished recognition of hypoglycaemic symptoms and reduced oral intake, which elevate the risk of ADEs. The use of immediate-acting or sliding-scale insulin is also discouraged in older adults living with dementia. Instead simplified and individualised treatment regimens are recommended with the aim of reducing treatment burden whilst still protecting against hyperglycaemia [18]. Although a decrease in rates of insulin supply were observed, we did not identify any participant factors associated with discontinuation. This highlights the possible need for education around discontinuation and dose reductions in those living with dementia approaching end-of-life [19].

Discontinuation was observed for metformin use in those with CKD, which is consistent with current guideline recommendation in the UK and Australia, whilst other glucose-lowering medication classes showed lower rates of discontinuation over the last year of life [4, 6]. This reflected similar findings from studies that observed a decrease in metformin use (44% in 12–9 months vs. 39% in 3–0 months of life) approaching end-of-life [20]. For other glucose-lowering medication, a slight increase in DPP4i use and no significant decrease of SGLT-2i or GLP-1ras have previously been reported [17]. Minimal discontinuation of other glucose-lowering medication may be due to lower initial supply rates, increased tolerability or lack of deprescribing recommendations in guidelines [21]. GLP-1ras, SGLT-2is and DPP-4is are often recommended for those at increased risk of hypoglycaemia as well as for their cardioprotective effects which may support their use in people living with dementia. However, recent studies report the need to consider frailty and co-morbidity status, such as dementia when deciding on use of GLP-1ras and SGLT2is due to risk of malnourishment or unexplained weight loss, dehydration, urinary incontinence and hypotension [22]. Therefore, careful re-evaluation of the potential harms of these medications is required in those living with dementia and limited life expectancy who are likely to be frail.

### Strengths and limitations

Employing the same methods in the UK and Australia enabled us to compare glucose-lowering medication use in patient populations. Single-country studies may not be designed with uniformity of cohorts and analysis methods in mind which can make cross-comparison challenging. Although cohorts were created to be as similar as possible, there were some variations between UK and Australian data. We analysed UK prescription data and Australian dispensing data. Selected co-morbidities were based on the most common recorded co-morbidities in those with T2D at death. The documented prevalence of CKD was lower in the UK cohort than in the Australian cohort (35.0% versus 64.5%). In UK and Australian population-based studies, CKD has been reported to be prevalent in around half of those aged 75 and over [23, 24]. Difference in cohort identification could partly explain the apparent difference in CKD prevalence. Previous studies in the CPRD-HES database have reported that using diagnosis codes alone may underestimate the ascertainment of CKD [25]. It is also possible that the hospital-based cohort in Australia had poorer renal function, although both cohorts had similar mean age and were limited to those in the final year of life. Age was defined differently across databases (age at first recorded T2D diagnosis in Australia and age at death in the UK). This reduces comparability between databases but is unlikely to affect the validity of regression analyses, which were conducted separately within each database. We were also unable to determine appropriateness of medication use at an individual level, or examine clinical parameters (e.g. HbA1c measurements), and therefore appropriateness of discontinuation was implied due to overall risk most likely outweighing benefits.

### Clinical and research implications

Investigating glucose-lowering medication supply patterns prior to death in UK and Australia provides insights into international variations in dementia and T2D care at the end-of-life. Our findings highlight that discontinuation occurred more in Australia and whilst both regions show signs of discontinuation, there is still need for practice and policy initiatives to optimise glucose-lowering medication use in the final year of life. The coexistence of dementia and T2D necessitates the need for medication management reviews and advance care planning, particularly as individuals approach the end-of-life. Advanced care planning is promoted in both the UK and Australia as an integral component of dementia care. In the UK, NICE guidelines recommend initiating advanced care planning early, ensuring that treatment preferences, including medication management and end-of-life decisions, reflect individual values and goals [3]. Similarly, Australia’s guidelines advocate proactive discussions, emphasising early involvement of patients, caregivers and interdisciplinary healthcare teams to clearly document and regularly update care preferences as dementia progresses [5, 6]. Despite guidelines in both countries recommending deprescribing or simplification of medication regimens at end-of-life, our findings highlight that glucose-lowering medication use often persists until death, underscoring a potential gap between clinical recommendations and practice or a reluctance to deprescribe. Our data reflects findings from qualitative studies; clinicians have reported that they are comfortable deprescribing these medications in those where harms clearly outweigh benefits; however, some expressed hesitancy when medications were started by a specialist [26, 27]. Studies show health care professionals need to feel confident that they are acting in the best interests of the individual and that they are equipped to meet their needs and that the system supports personalised care [28]. Structured frameworks such as the 4S pathway for realigning diabetes regimens in older adults [29] can help ensure regimens remain safe for the individual. Improved teamwork approaches such as multidisciplinary case reviews could assist clinicians to feel supported initiating deprescribing [30] and electronic health record alerts could be implemented for people living with dementia in aged care settings who use glucose-lowering medication to prompt timely medication management reviews [31].

## Conclusion

Despite observing discontinuation of glucose-lowering medications in those living with dementia, 56.5–82.8% of individuals were supplied diabetes mediations in the final 3 months of life. Our findings reveal that glucose-lowering medication use often persists until death in people living with dementia and T2D in the UK and Australia. To safeguard comfort and minimise harm in this population, structured, patient-centred deprescribing pathways should form an integral component of T2D management in dementia care.

## Supplementary Information

Below is the link to the electronic supplementary material.

Supplementary file1 (DOCX 29 KB)

## Data Availability

The data that support the findings of this study are available on request from the corresponding author. The data are not publicly available due to privacy or ethical restrictions.
