# Supplementary material for: Diabetes medication use in the last year of life in people living with dementia in the United Kingdom and Australia
Source: Eur Geriatr Med. 2026 Sep 27;17(5):2475–83. doi: 10.1007/s41999-026-01608-6 (PMC13755013; doi:10.1007/s41999-026-01608-6)
Supplement: Supplementary file 1 [file 41999_2026_1608_MOESM1_ESM.docx]

**Supplementary Information**

Supplementary Table 1a. ICD-10 codes for co-morbidities

| Disease | ICD-10 |
| --- | --- |
| Cardiovascular Disease | I00-I99 (excluding I84 – haemorrhoids) |
| - Acute rheumatic fever and Rheumatic heart disease | I00–I09 |
| - Hypertensive disease | I10–I15 |
| - Coronary heart disease | I20–I25 |
| - Angina | I20 |
| - Acute myocardial infarction | I21 |
| - Atrial fibrillation and futter | I48 |
| - Heart failure and cardiomyopathy | I50, I25.5, I42.0, I42.5–I42.9, I43 |
| - Heart failure | I50 |
| - Cerebrovascular disease | I60–I69 |
| - Stroke | I60–I64 |
| - Peripheral arterial disease | I70–I74 |
| - Atherosclerosis of peripheral arteries | I70.2 |
| - Abdominal aortic aneurysm | I71.3–I71.4 |
| - Transient ischaemic attack | G45 |
| - Congenital heart disease | Q20–Q26 |
| Chronic kidney disease |  |
| - Diabetic nephropathy | E10.2, E11.2, E13.2, E14.2 |
| - Hypertensive kidney disease | I12, I13, I15.0, I15.1 |
| - Glomerular diseases | N00–N08 |
| - Kidney tubulo-interstitial diseases | N11, N12, N14, N15, N16 |
| - Chronic kidney failure | N18 |
| - Unspecified kidney failure | N19 |
| - Other disorders of kidney and ureter | N25–N28, N391, N392, D59.3, B52.0, E85.3 |
| - Congenital malformations | Q60–Q63 |
| - Complications related to dialysis and kidney transplant | T82.4, T86.1 |
| - Preparatory care for dialysis | Z49.0 |
| - Kidney transplant and dialysis status | Z94.0, Z99.2 |
| - Supplementary code for CKD stage 3-5, which maps to ICD-10-AM N18.3, N18.4 and N18.5 | U87.1 |
| Cancer |  |
| - All malignant cancers | C00-C97 |

Supplementary Table 1b.. Glucose lowering medication classes code list

| (ATC code) Medication Class | Medication PBS Codes (AUS) | BNF codes (UK) |
| --- | --- | --- |
| A10A Insulin | A10AB - Insulins and analogues for injection, fast-acting  A10AC - Insulins and analogues for injection, intermediate-acting  A10AD - Insulins and analogues for injection, intermediate- or long-acting combined with fast-acting  A10AE - Insulins and analogues for injection, long-acting  A10AF - Insulins and analogues for inhalation | Biphasic insulin aspart (0601012W0)  Biphasic insulin lispro (0601012F0)  Biphasic isophane insulin (0601012D0)  Insulin degludec (0601012Z0)  Insulin detemir (0601012X0)  Insulin glargine (0601012V0)  Insulin glargine/lixisenatide (0601012AB)  Insulin human (0601011R0)  Insulin zinc suspension (0601012G0)  Isophane insulin (0601012S0)  Protamine zinc insulin (0601012U0)  Insulin Aspart (0601011A0)  Insulin Glulisine (0601011P0)  Insulin Lispro (0601011L0)  Insulin Neutral Human (0601011N0) |
| A10BA Biguanides | A10BA02 metformin | Metformin (0601022B0) |
| A10BB Sulfonylureas | A10BB01 glibenclamide  A10BB07 glipizide  A10BB09 gliclazide A10BB12 glimepiride | Chlorpropamide (0601021E0)  Tolbutamide (0601021X0)  Glibenclamide (0601021H0)  Gliclazide (0601021M0)  Glimepiride (0601021A0)  Glipizide (0601021P0) |
| A10BD Combination of oral blood glucose lowering drugs *(to be included in code for each corresponding medication class)* | A10BD07 metformin and sitagliptin  A10BD08 metformin and vildagliptin  A10BD10 metformin and saxagliptin  A10BD11 metformin and linagliptin  A10BD13 metformin and alogliptin A10BD15 metformin and dapagliflozin  A10BD19 linagliptin and empagliflozin  A10BD20 metformin and empagliflozin  A10BD21 saxagliptin and dapagliflozin | Alogliptin + Metformin 0601023AJ  Dapagliflozin + Metformin 0601023AL  Empagliflozin + Linagliptin 0601023AY  Empagliflozin + Metformin 0601023AR  Ertugliflozin + Sitagliptin  Linagliptin + Metformin 0601023AF  Saxagliptin + Dapagliflozin 0601023AV  Saxagliptin + Metformin 0601023AH  Sitagliptin + Metformin 0601023AD  Vildagliptin + Metformin 0601023Z0 |
| A10BG – Thiazolidinediones | A10BG03 pioglitazone | Rosiglitazone (0601023S0)  Pioglitazone (0601023B0) |
| A10BH - Dipeptidyl peptidase 4 (DPP-4) inhibitors | A10BH01 sitagliptin  A10BH02 vildagliptin  A10BH03 saxagliptin  A10BH04 alogliptin  A10BH05 linagliptin | Sitagliptin 0601023X0  Vildagliptin 0601023AA  Alogliptin 0601023AK  Linagliptin 0601023AE  Saxagliptin 0601023AC |
| A10BJ - Glucagon-like peptide-1 (GLP-1) analogues | A10BJ01 exenatide  A10BJ05 dulaglutide  A10BJ06 semaglutide | Exenatide (0601023Y0)  Liraglutide (0601023AB)  Lixisenatide (0601023AI)  Albiglutide (0601023AS)  Dulaglutide 0601023AQ  Semaglutide 0601023AW |
| A10BK - Sodium-glucose co-transporter 2 (SGLT2) inhibitors | A10BK01 dapagliflozin  A10BK03 empagliflozin | Dapagliflozin 0601023AG  Empagliflozin 0601023AN  Ertugliflozin 0601023AX  Canagliflozin (0601023AM) |
| Other |  | Ins degludec/liraglutide (0601023AU)  Nateglinide (0601023U0)  Repaglinide (0601023R0) |

Supplementary Table 1c. Dementia medication code list

| **(ATC code) Dementia drugs** | **AUS** | **UK** |
| --- | --- | --- |
| (N06DA) Anticholinesterases | N06DA02 donepezil  N06DA03 rivastigmine  N06DA04 galantamine | Donepezil hydrochloride (0411000D0)  Galantamine (0411000F0)  Idebenone (0411000H0)  Rivastigmine (0411000E0) |
| (N06DX) Other anti-dementia drugs | N06DX01 memantine | Memantine hydrochloride (0411000G0)  Lecanemab and donanemab |

Supplementary Table 2. Prevalence of glucose-lowering medication class use prior to death

| Medication Class | Within 12 Months | | | | Within 9 Months | | | | Within 6 Months | | | | Within 3 Months | | | |
| --- | --- | --- | --- | --- | --- | --- | --- | --- | --- | --- | --- | --- | --- | --- | --- | --- |
|  | **Australia** | | **UK** | | **Australia** | | **UK** | | **Australia** | | **UK** | | **Australia** | | **UK** | |
|  | **N** | **% of overall population** | **N** | **% of overall population** | **N** | **% of 12-month users** | **N** | **% of 12-month users** | **N** | **% of 12-month users** | **N** | **% of 12-month users** | **N** | **% of 12-month users** | **N** | **% of 12-month users** |
| Metformin | 2,637 | 41.0 | 3,633 | 36.0 | 2,499 | 94.8 | 3,488 | 96.0 | 2,354 | 89.3 | 3,300 | 90.8 | 2,016 | 76.5 | 2,984 | 82.1 |
| Insulin | 2,175 | 33.8 | 1,965 | 19.5 | 2,012 | 92.5 | 1,908 | 97.1 | 1,732 | 79.6 | 1,785 | 90.8 | 1,228 | 56.5 | 1,462 | 74.4 |
| Sulfonylureas | 1,797 | 27.9 | 2,179 | 21.6 | 1,682 | 93.6 | 2,064 | 94.7 | 1,530 | 85.1 | 1,925 | 88.3 | 1,274 | 70.9 | 1,669 | 76.6 |
| Other glucose-lowering medications | 970 | 15.1 | 1,133 | 11.2 | 913 | 94.1 | 1,094 | 96.6 | 837 | 86.3 | 1,041 | 91.9 | 742 | 76.5 | 938 | 82.8 |

Supplementary Table 3. Rates of medication supply in individuals supplied glucose-lowering medication

|  | Australia | | UK | |
| --- | --- | --- | --- | --- |
| Medication class | **No. supplied at 12-6 months** | **No. discontinued supply at 6 – 0 months (%)** | **No. supplied at 12-6 months** | **No. discontinued supply at 6 – 0 months (%)** |
| Metformin | 2,462 | 283 (11.5) | 3,534 | 333 (9.4) |
| Insulin | 1,645 | 443 (26.9) | 1,793 | 180 (10.4) |
| Sulfonylurea | 1,667 | 267 (16) | 2,059 | 254 (12.3) |
| Other glucose-lowering medications (DPP4i, SLT2i, GLP-1ra, TZDs) | 834 | 133 (15.9) | 969 | 92 (9.5) |
